# Gunshot Wound in Lumbar Spine with Intradural Location of a Bullet

**DOI:** 10.1155/2014/698585

**Published:** 2014-06-04

**Authors:** G. Bordon, S. Burguet Girona

**Affiliations:** Department of Orthopedic Surgery, Hospital de Manises, Avenida Generalitat Valenciana 50, Manises, 46940 Valencia, Spain

## Abstract

The presence of a migratory bullet in the spinal canal after a gunshot injury is a rare finding, specially without causing permanent neurologic damage. We present the case of a patient who suffered a gunshot wound with an entry point in the posterior arc of L2-L3 and a migratory bullet detected at the level of L5-S1 in the CT scan. The patient complained about intense headache, dizziness, and variable sensitive impairment in lower legs apparently depending on the patient's position in bed. We decided to remove the bullet in order to prevent the delayed neurological damage and lead toxicity. We discuss technical details of this surgery.

## 1. Introduction

Firearm injuries, although infrequent, are on the rise in our environment. The presence of a bullet in the subdural space without causing neurological injuries is a rare scenario.

The management of these patients might be controversial. We present the case of a patient who suffered a gunshot wound where the bullet entered the spinal canal at the posterior arc of L2-L3 and migrated to the lowest point of the spinal canal without causing neurological damage. We comment on the surgical technique with special reference to the manoeuvres necessary for trapping the bullet during surgery.

## 2. Case Report

**Figure 1:**
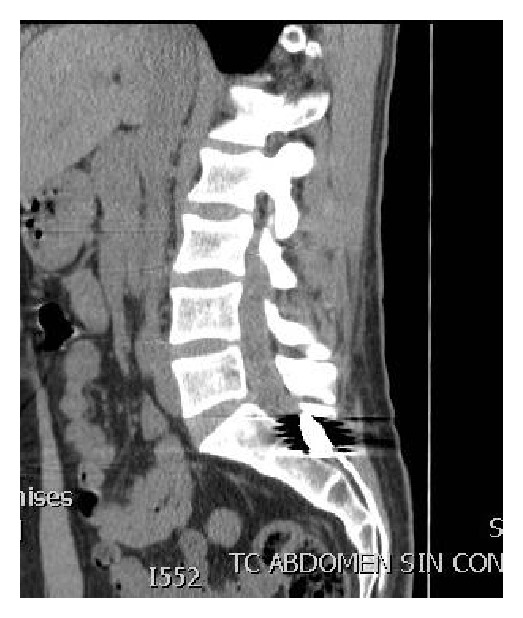
Sagital CT scan shows the bullet lodged at the S2 level.

**Figure 2:**
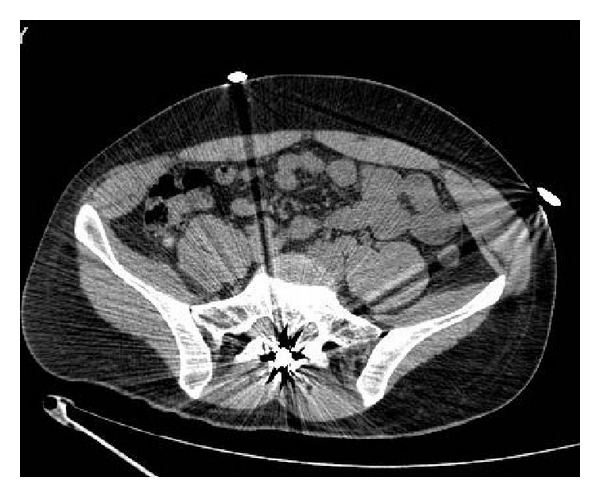
Axial CT scan demonstrates the intracanal position of the bullet and no damage to the posterior arc of S1-S2 level.

A 27-year-old patient was referred to our spine center after suffering from multiple gunshot wounds. At the first receiving institution, two bullets were removed from thorax and thigh muscle and after initial stabilization the patient was referred to our institution. Computed tomography scan of spine showed a third bullet located apparently in the spinal canal at the level of S1-S2 (Figures 1 and 2).

**Figure 3:**
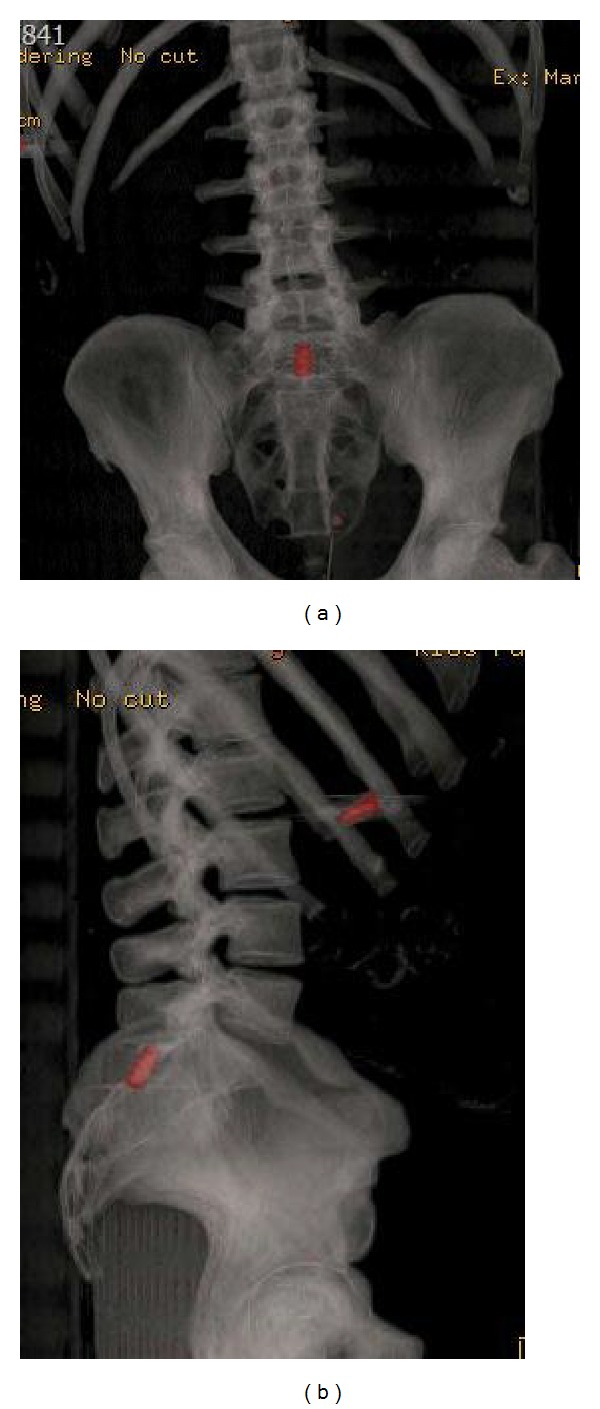
3D CT scan reconstruction.

The patient was a habitual drug consumer of cannabis and cocaine. After initial exploration, no entry wound could be detected at the level of S1-S2. The only wound compatible with entry hole of the bullet in the spinal canal was detected approximately 10 cm lateral to the midline at the level of L2. After neurological exploration, the only impairment found on reception of the patient was hypoesthesia at the perianal area and on the inside of both thigh muscles corresponding to the sacral roots. No muscle weakness was detected on exploration and sphincter function seemed to be normal. The CT scan showed a bullet at the levels S1 and S2 inside the spinal canal, but no damage to the posterior arc could be visualized at this level (Figures 3(a) and 3(b)).

**Figure 4:**
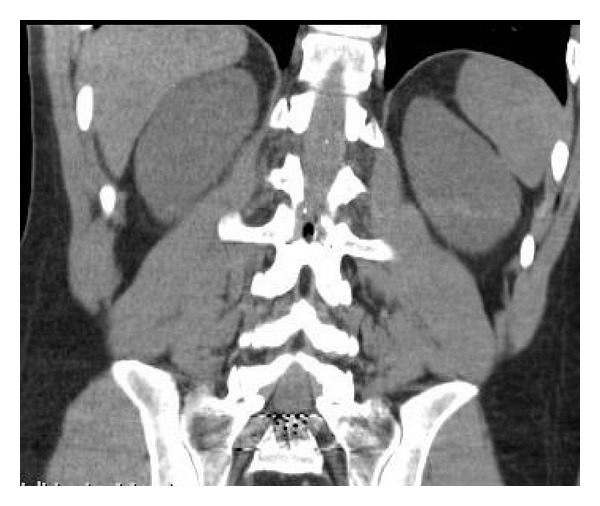
In the coronal CT scan we can see the damage to the posterior arc of L2 that was the entry point of the bullet.

The only damage to the posterior arc compatible with an entry hole for the bullet was seen at the posterior arc of L2 level (Figure 4). The patient complained about intense headache and dizziness which got worse since the reception and variable sensitive impairment in lower legs apparently depending on the patient's position in bed. No cerebrospinal fluid (CSF) leakage was evident.

**Figure 5:**
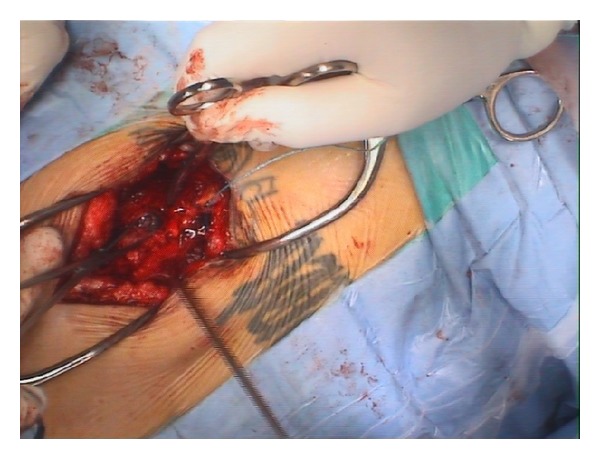
Intraoperative image of the bullet inside the spinal canal before the removal.

After hemodynamic stabilization, the patient was operated on the 4th day after reception. No leakage of CSF was observed at the possible entry hole at the L2 level. A posterior approach to L5/S1 level with laminotomy of L5 and S1 was performed. After positioning of the patient in a prone position on the surgical table the fluoroscopic control showed a change in position of the bullet. It moved to the lowest point of the spinal canal just posterior to L5. After exposure of the dural sac the bullet was located with fluoroscopy and free floating of the bullet in the dural sac was observed after minimal manipulation with Kerrison Rongeurs during laminotomy. The operating table was tilted to a position where the pelvis was lower than lumbar spine (reverse Trendelenburg's position) to help for a caudal migration of the bullet by gravity. To be able to extract the bullet it had to be trapped with a blunt dissector to prevent it from ascending in the dural canal (Figure 5).

After observing the silhouette of the bullet through the transparent dura a midline durotomy was performed to expose the intrathecal bullet, which was removed. The dura was sutured with 6-0 nylon thread and collagen glue (Tissucol) was applied for watertight closure. During postoperative care after 2 days of bed rest the patient was mobilized without problems. The patient kept complaining about intense headache after surgery and was treated with common analgesics. No evidence of postoperative CSF leakage was found. The hypoesthesia improved and no additional neurological damage was detected after surgery. The patient received antibiotic prophylaxis during 7 days after surgery. Headache and paresthesias were resolved completely after 3 weeks.

## 3. Discussion

We present this case because the intrathecal localization and migration of a bullet without causing permanent neurological damage are even less frequent and to our knowledge only one case with similar characteristics has been reported [1].

Actually, there is no specific protocol available for treatment decision in gunshot spinal cord injuries, like in blunt and penetrating chest and abdominal trauma [2].

Several studies have shown that patients do not benefit from the use of steroids in penetrating wounds to the spinal canal and in this case they were not used either [3, 4].

Surgical treatment should be individualized and during the decision finding process for surgery several facts have to be taken into account such as neurological damage, bullet location, spinal stability, associated injuries, and hemodynamic stability. We found controversy about decompression surgery in complete and incomplete gunshot spinal cord injuries, as in spinal cord injuries due to other causes [2].

The migration of intradurally located bullet has been described in literature before [2, 5–10]. In most cases the migration has been following gravity and away from the medullary cone to the lowest place in the spinal canal at the lumbosacral area where the spinal canal is narrowing and trapping the bullet at this location.

This might explain why in this case the supposedly heat irradiating bullet, when entering the spinal canal close to the medullary cone, did not cause any medullary lesion. Migration further distally inside the dural sac did not cause further nerve root damage either and the only nervous symptoms noted were caused by irritation of the lumbar and sacral nerve roots at the final stop of the bullet.

We decided to perform surgery in this case when the patient was hemodynamically stable.

The bullet was of easy accessibility in the sacrolumbar junction and by removing it we pretended to avoid future nerve root impairment or lead toxicity possibly caused by a free floating bullet in the spinal canal, like what other authors recommend [1, 6, 11].

During surgery the surgeon has to be prepared to extract a foreign body, which is moving inside the dural sac. Since the bullet is moving inside the dural sac a strategy has to be used in order to trap it first and then remove it; otherwise, minimal manipulation with the instruments might cause cranial migration of the bullet and unnecessary extension of laminotomy and/or durotomy. Positioning of the surgical table in a head-up and leg-down position (reverse Trendelenburg's position) and trapping the bullet with a blunt dissector helped to be able to catch and remove the bullet in this case.

## 4. Conclusion

We agree with other authors that the presence of a migratory bullet in the spinal canal is an indication of surgery, to avoid the possibility of delayed neurological symptoms and the theoretical lead toxicity. The patient in a reverse Trendelenburg's position and trapping the bullet with a blunt dissector are useful tricks to facilitate catching the floating bullet.
